# Supplementary material for: Palmitoylation targets the calcineurin phosphatase to the phosphatidylinositol 4-kinase complex at the plasma membrane
Source: Nat Commun. 2021 Oct 18;12:6064. doi: 10.1038/s41467-021-26326-4 (PMC8523714; doi:10.1038/s41467-021-26326-4)
Supplement: Supplementary file 1 — Supplementary Information [file 41467_2021_26326_MOESM1_ESM.pdf]

Supplementary Information for

Palmitoylation targets the Calcineurin phosphatase to the Phosphatidylinositol 4-kinase complex at the plasma membrane

Idil Ulengin-Talkish[1], Matthew AH Parson[2], Meredith L Jenkins[2] Jagoree Roy[1], Alexis ZL Shih[3*], Nicole St-Denis[4^], Gergo Gulyas[5], Tamas Balla[5], Anne-Claude Gingras[4,6], Péter Várnai[7], Elizabeth Conibear[3], John E Burke[2,8], Martha S. Cyert[1, #]

1. Department of Biology, Stanford University, Stanford CA
2. Department of Biochemistry and Microbiology, University of Victoria BC, Canada
3. Department of Medical Genetics, University of British Columbia, Vancouver, Canada
4. Lunenfeld-Tanenbaum Research Institute at Mount Sinai Hospital, University of Toronto, Toronto, Canada
5. Section on Molecular Signal Transduction, National Institute of Child Health and Human Development, National Institutes of Health, Bethesda, MD
6. Department of Molecular Genetics, University of Toronto, Toronto, ON, Canada
7. Department of Physiology, Faculty of Medicine, Semmelweis University, Budapest, Hungary
8. Department of Biochemistry, The University of British Columbia, Vancouver BC, Canada

* Present address: Max-Delbrueck Center for Molecular Medicine, Berlin, Germany

^ Present address: High-Fidelity Science Communications, Summerside, PE, Canada

\# Corresponding author

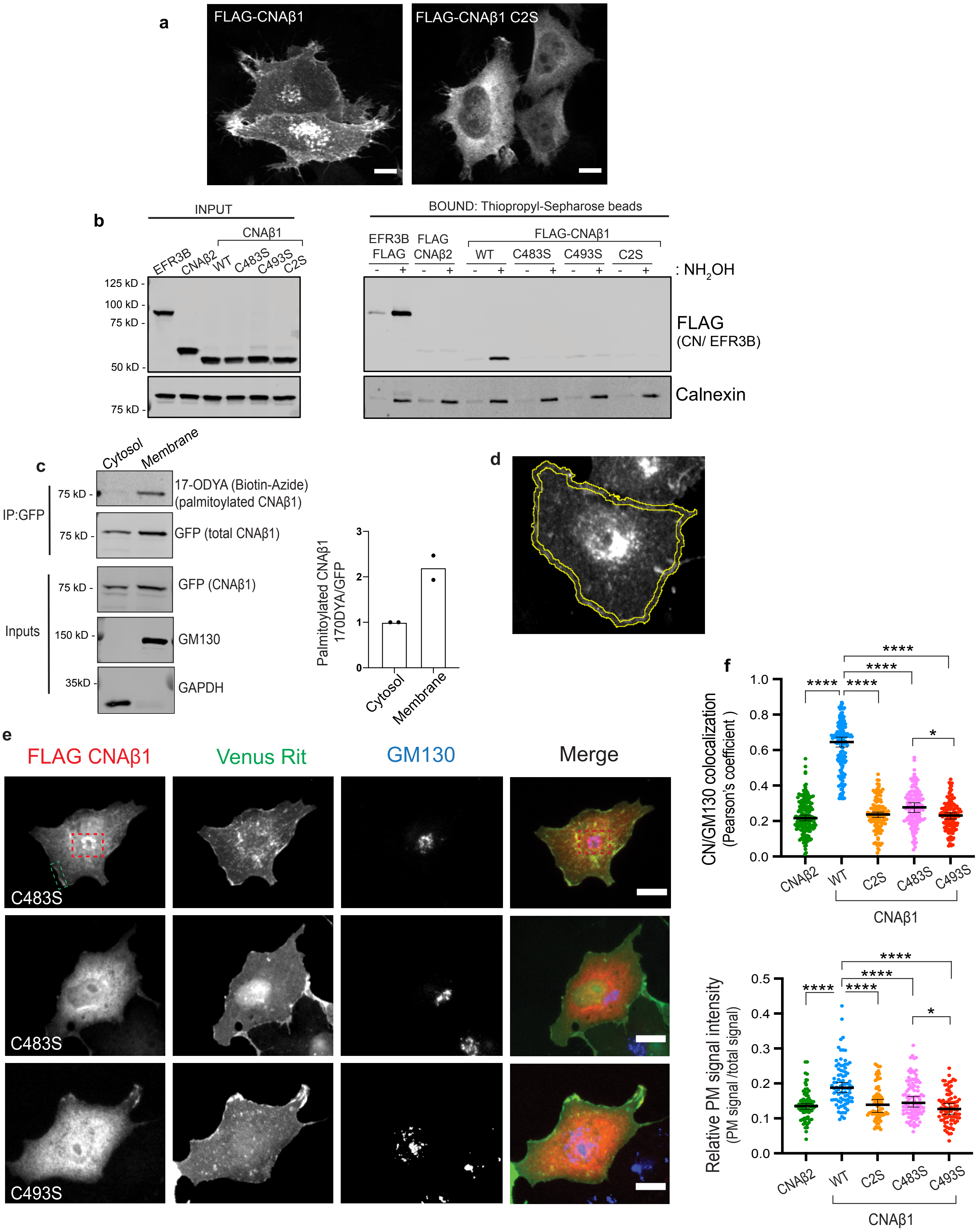

Supplementary Figure 1: CNAβ1 localizes to membranes via palmitoylation. **a** Representative images of HeLa cells expressing FLAG-CNAβ1 WT or CNAβ1 C2S. Scale bar = 15 μm. (≥ 70 cells from 3 independent experiments). **b** Analysis of palmitoylation via Acyl-RAC of COS-7 cells expressing FLAG-CNAβ2, FLAG-CNAβ1 (WT or cysteine mutants) or EFR3B-FLAG; immunoblot representative of 3 independent experiments. **c** Fractionation of GFP-CNAβ1 expressed in COS-7 cells labelled with 17-ODYA to detect palmitoylation. 17-ODYA visualized on immunoblot using streptavidin (SA) following CLICK chemistry with azide-Biotin. CNAβ1 palmitoylation quantified by SA signal (17-ODYA) / total protein signal (GFP). Data presented as mean values across 2 independent experiments. **d** Representative image showing 5-pixel wide region of interest (ROI) at cell periphery defining PM in image analyses (Fig.1f and Supplementary Fig. 1f). **e** Representative images of COS-7 cells expressing FLAG-CNAβ1 C483S (top 2 rows) or C493S (bottom row) with Venus-RIT (PM, green) immunostained with anti-FLAG (red) and anti-GM130 (Golgi, blue). Top row shows representative cell with weak PM (green box) and Golgi (red box) localization. Scale bar = 15 μm. **f** Top: co-localization of indicated FLAG-CNAβ's with GM130 using Image J (EZcolocalization plug-in); median Pearson`s coefficients with 95% confidence intervals (CI) shown. ≥ 100 cells from 3 independent replicates analyzed. * p =0.0141, **** p <0.0001 Bottom: anti-FLAG intensity at cell periphery (as in **d**) normalized to total intensity; median with 95% CI shown. ≥ 70 cells from 3 independent replicates analyzed. * p =0.0279, **** p <0.0001. One- way ANOVA followed by Kruskal-Wallis test used for both analyses.

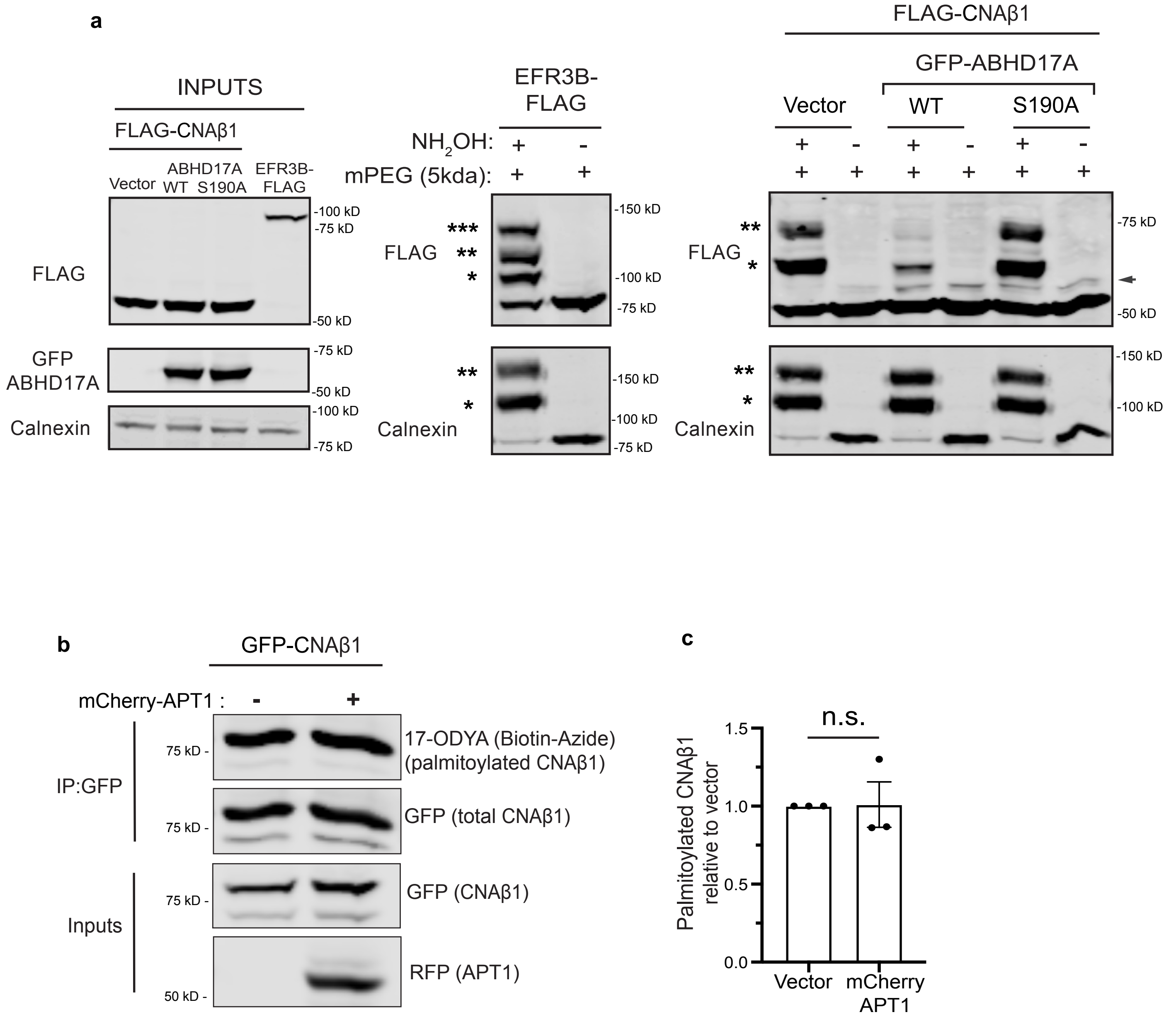

Supplementary Figure 2: CNAβ1 is depalmitoylated by ABHD17A but not by APT1: **a** Representative immunoblot of Acyl-PEG exchange assay done in COS-7 cells co-expressing FLAG-CNAβ1 with vector, GFP-ABHD17A WT or GFP-ABHD17A catalytic mutant, S190A. The number of PEGylation (reflecting S-palmitoylation) events are indicated by asterisks (*). Arrowhead indicates non-specific antibody band. n =2 independent experiments. **b** GFP-CNAβ1 palmitoylation levels in COS-7 cells co-expressing vector or mcherry-APT1 determined using metabolic labelling with 17-ODYA. 17-ODYA visualized on immunoblot using streptavidin (SA) following CLICK chemistry with azide-Biotin. **c** GFP-CNAβ1 palmitoylation levels (in b) are quantified by the SA signal (17-ODYA) / total protein signal (GFP) normalized to vector control. Data are mean ± SEM (n=3 independent experiments); n.s. not significant (p =0.94) using unpaired, two tailed t-test.

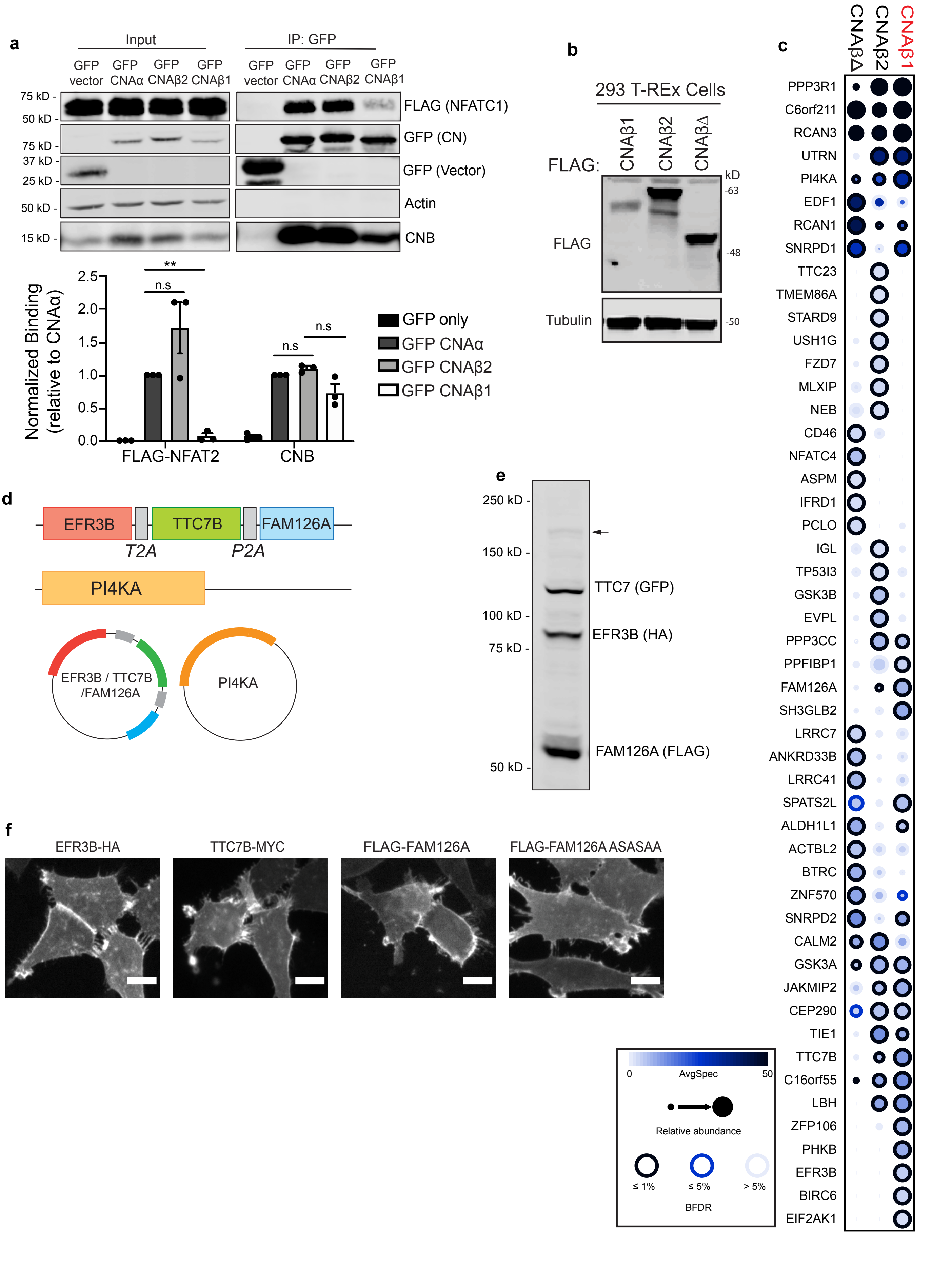

Supplementary Figure 3: AP-MS analyses with CNAβ baits and expression of PI4KA complex. **a** Immunoblot of anti-GFP immunoprecipitates from HEK T-Rex lines expressing GFP, GFP-CNAα, GFP-CNAβ2 or GFP-CNAβ1, transfected with FLAG-NFATC1. Co-purification quantified for FLAG-NFATC1 and CNB as = bound FLAG or anti-CNB signal / bound GFP signal normalized to input. Mean ± SEM shown for n=3 independent experiments, relative to GFP-CNAα. n.s. not significant ($p > 0.4$), ** $p = 0.0044$ using one-way ANOVA with Dunnette's multiple comparison tests. **b** Immunoblot of FLAG-CNβ bait expression in HEK T-Rex lines used for AP-MS. **c** Complete dotplot of CN-interactors identified by AP-MS; legend shows colors for BFDR (Bayesian False Discovery Rate). **d** Schematic of plasmid encoding EFR3B-HA (red), TTC7B-MYC (or GFP, green) and FLAG-FAM126A (blue) separated by 2A viral peptides, T2A and P2A (gray); combined with PI4KA (orange) plasmid when indicated. **e** Immunoblot of HeLa cells expressing plasmid in d, arrow: residual uncut P2A form. n ≥10 independent experiments. **f** Representative HeLa cells transfected with plasmid in d immunostained with anti-HA, anti-MYC or anti-FLAG. Scale bar = 15 µm. Images are representative of ≥ 100 cells from 2 independent experiments.

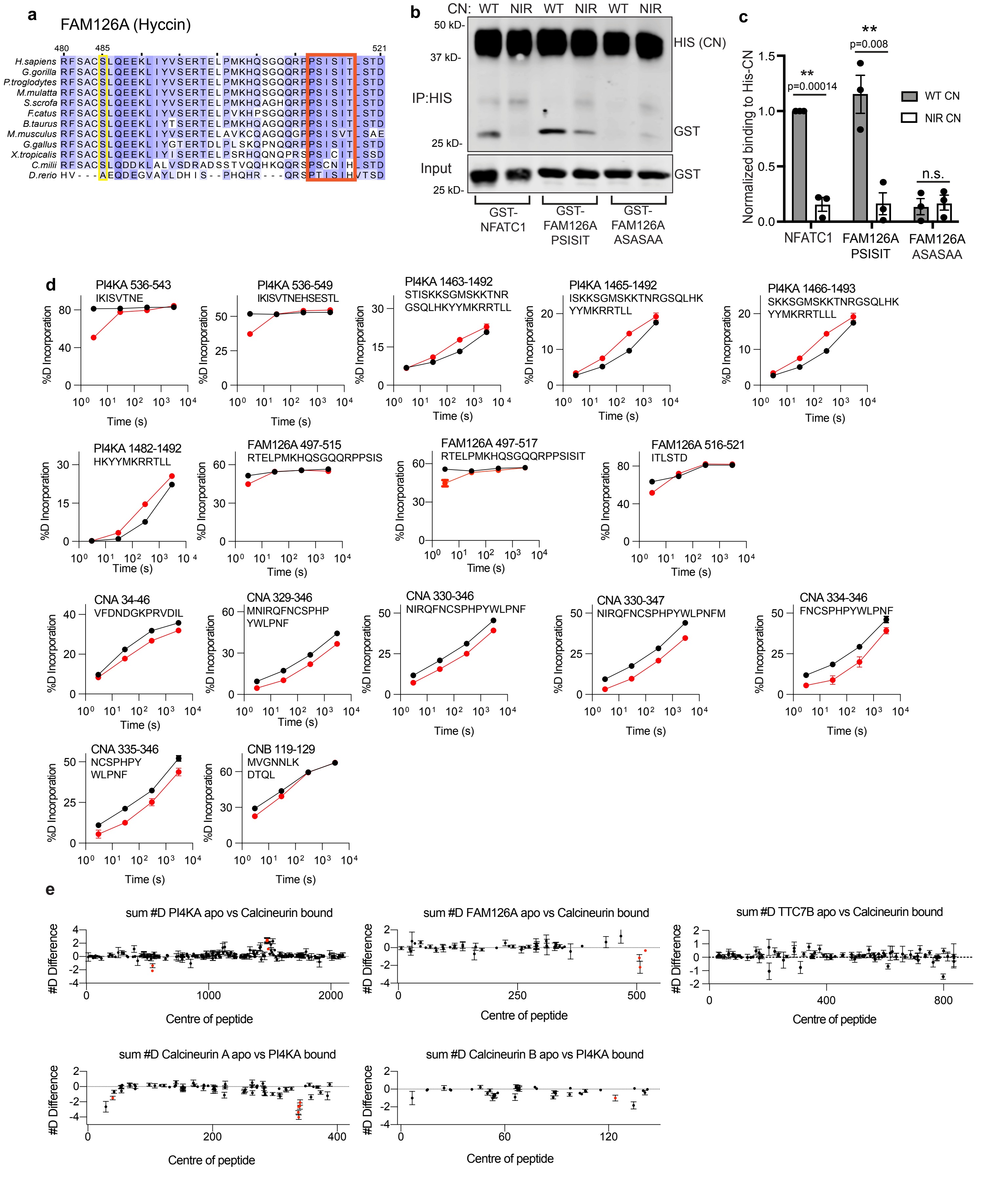

Supplementary Figure 4: CN interactions with PI4KA complex. **a** Sequence conservation of the FAM126A C-terminus (a.a 480-521) across vertebrates. PxIxIT motif (red) and phosphorylated serine 485 (yellow) are boxed. **b** Immunoblot (representative of 3 independent experiments) showing co-purification with His-CN (WT or NIR mutant) of GST-tagged peptides with PxIxIT motifs from NFATC1, FAM126A WT (SGQQRPPSISITLSTD) or mutant (SGQQRPASA-SAALSTD). **c** Quantification of co-purifications in b. Mean ± SEM; for n=3 independent experiments is shown. For NFATC1 (WT vs NIR) p =0.00014, for FAM126A PSISIT(WT vs NIR) p =0.008 calculated using multiple unpaired t-tests, n.s. not significant. **d** Deuterium incorporation for all peptides showing significant increase or decrease in exchange in CN-PI4KA trimer complex (>5%, 0.4 Da, and an unpaired, two-tailed t-test p <0.01) (lines: black, without and red, with co-incubation). For all panels, error bars show ± SD (n = 3 independent experiments). **e** The number of HDX for all analyzed peptides over the entire time course. Each point represents the central residue of an individual peptide. Significant peptides are highlighted in red. Error bars show ± SD (n=3 independent experiments). Raw data and statistical analyses for each analyzed peptide provided in Source Data file.

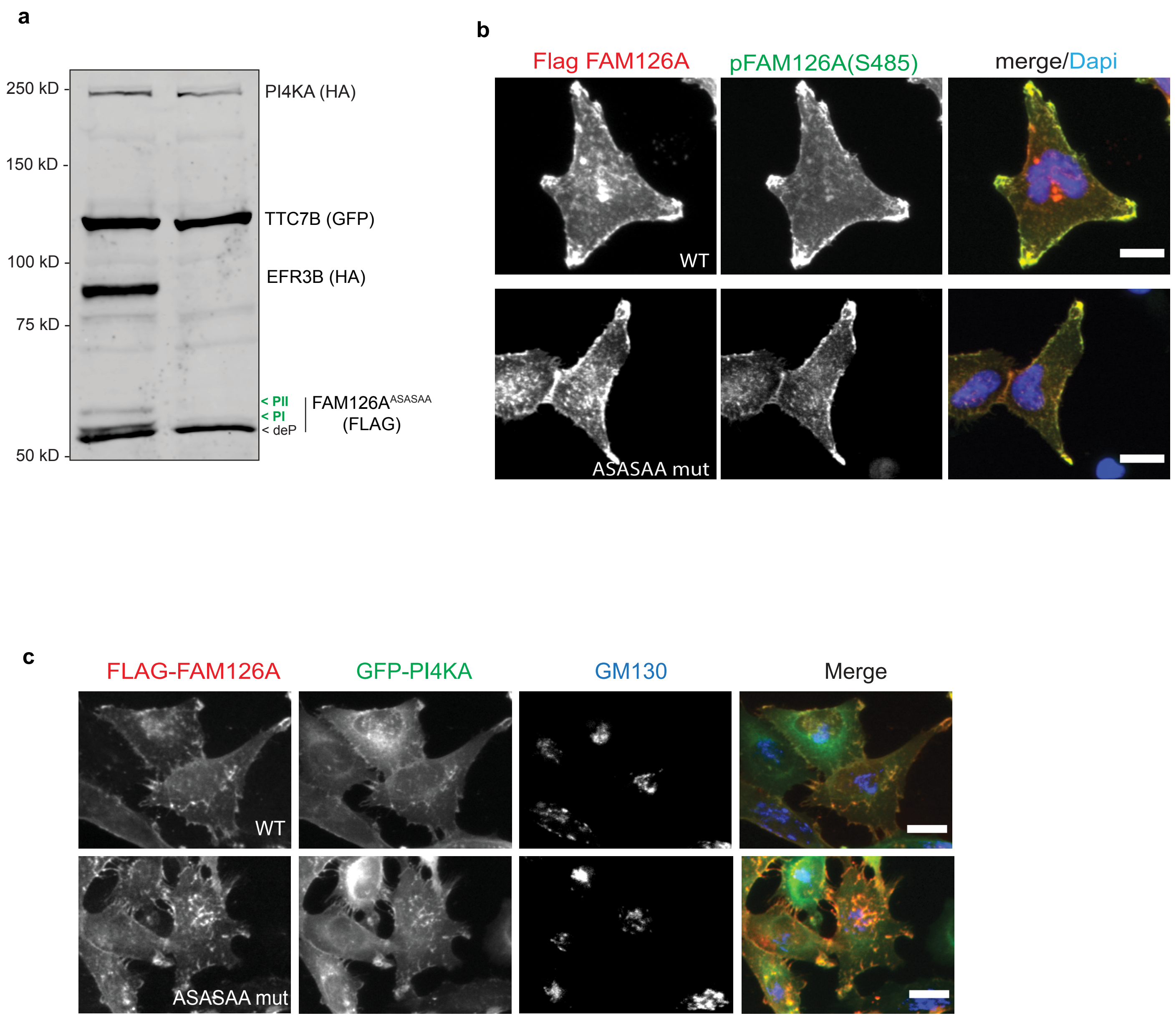

Supplementary Figure 5: Phosphorylation of FAM126A occurs at the PM. **a** Representative immunoblot showing electrophoretic mobility shifts in FAM126A ASASAA mutant in lysates of HeLa cells co-expressing HA-PI4KA and TTC7B-GFP in the presence or absence of EFR3B-HA. PI and PII denote phosphorylated forms, deP denotes dephosphorylated FAM126A (n = 3 independent experiments). **b** Representative images of fixed HeLa cells transfected with EFR3B HA_T2A_TTC7 MYC_P2A_ FLAG FAM126A (WT or ASASAA) plasmids, stained with anti-FLAG (red), anti-pFAM126A S485 (green) antibodies and DAPI (blue). Scale bar = 15 µm (representative of ≥ 20 cells from 2 independent experiments). **c** Images showing localizations of GFP-PI4KA (green) and FLAG-FAM126A (WT or ASASAA mutant) in COS-7 cells co-expressing EFR3B-HA, TTC7B-MYC. Representative images of cells fixed and immunostained with anti-FLAG (red) and anti-GM130 (blue) antibodies are shown. Scale bar = 15 µm. Images are representative of ≥ 100 cells from 2 independent experiments.

Supplementary Table 1: HDX-MS data analysis statistics summary.

Protein Data Set	PI4KA	FAM126A	TTC7B	Calcineurin A	Calcineurin B
HDX reaction details	%D2O=65% pH(read)= 7.5 Temp= 18°C	%D2O=65% pH(read)= 7.5 Temp= 18°C	%D2O=65% pH(read)= 7.5 Temp= 18°C	%D2O=65% pH(read)= 7.5 Temp= 18°C	%D2O=65% pH(read)= 7.5 Temp= 18°C
HDX time course	3s, 30s, 300s, 3000s	3s, 30s, 300s, 3000s	3s, 30s, 300s, 3000s	3s, 30s, 300s, 3000s	3s, 30s, 300s, 3000s
HDX controls	N/A	N/A	N/A	N/A	N/A
Back-exchange	Corrected based on %D2O	Corrected based on %D2O	Corrected based on %D2O	Corrected based on %D2O	Corrected based on %D2O
Number of peptides	234	53	106	81	40
Sequence coverage	77.6%	80.9%	84.2%	89%	89.3%
Average peptide length / Redundancy	Length = 13.3 Redundancy = 1.3	Length = 14.4 Redundancy = 1.3	Length = 12.3 Redundancy = 1.3	Length = 11.5 Redundancy = 2.3	Length = 14.0 Redundancy = 3.2
Replicates	3 (3s apo and 30s complex in duplicate)	3 (3s apo and 30s complex in duplicate)	3 (3s apo and 30s complex in duplicate)	3 (30s complex in duplicate)	3 (30s complex in duplicate)
Repeatability	Average StDev = 0.7%	Average StDev = 0.8%	Average StDev = 0.6%	Average StDev = 0.8%	Average StDev = 0.6%
Significant differences in HDX	>5% and >0.5 Da and unpaired, two-tailed t-test p<0.01	5% and >0.5 Da and unpaired, two-tailed t-test p<0.01	5% and >0.5 Da and unpaired, two-tailed t-test p<0.01	5% and >0.5 Da and unpaired, two-tailed t-test p<0.01	5% and >0.5 Da and unpaired, two-tailed t-test p<0.01

Supplementary Table 2: Primer list used in this study.

PRIMERS	OLIGONUCLEOTIDE SEQUENCE (5`->3`)	SOURCE
FAM126A_PSISIT->ASASAA quickchange Forward:	cagagacctgctagtgctagcgctgctctgtccacagat	This study
FAM126A_PSISIT->ASASAA quickchange Reverse:	atctgtggacagagcagcgctagcactagcaggtctctg	This study
FAM126A 16-mer PxIxIT (PSISIT) containing peptide Forward:	gatccagcggccagcagcgcccgccgagcattagcattaccctgagcaccgattagc	This study
FAM126A 16-mer PSISIT containing peptide Reverse:	tcgagctaatcggtgctcagggtaatgctaatgctcggcgggcgctgctggccgctg	This study
FAM126A 16-mer ASASAA mutant peptide Forward:	gatccagcggccagcagcgcccggcgagcgcgagcgcggcgctgagcaccgattagc	This study
FAM126A 16-mer ASASAA mutant peptide Reverse:	tcgagctaatcggtgctcagcgccgcgctcgcgctcgccgggcgctgctggccgctg	This study
NFATC1 16-mer PxIxIT peptide Forward:	gatcccctgccctggagagtcctcgcatcgagataacctcgtgcttgggcctgtaatagc	Wigington et al., 2020 [1]
NFATC1 16-mer PxIxIT peptide Reverse:	tcgagctattacaggcccaagcacgaggttatctcgatgcgaggactctccagggcaggg	Wigington et al., 2020 [1]
FAM126A_S485A quickchange Forward:	aggttttccgcttgtgctctccaagaagaaaagc	This study
FAM126A_S485A quickchange Reverse:	gcttttcttcttggagagcacaagcggaaaacct	This study
T2A sequence	ggaagcggagagggcagaggaagtctgctaacatgcggtgacgtcgaggagaatcctggacct	This study
P2A sequence	ggaagcggagctactaacttcagcctgctgaagcaggctggagacgtggaggagaaccctggacct	This study
CNAβ1_C483S quickchange Forward:	ctcttttgctaacaattcacataatgcaagcagggaattccttc	This study
CNAβ1_C483S quickchange Reverse:	gaaggaattccctgcttgcattatgtgaattgttagcaaaagag	This study
CNAβ1_C493S quickchange Forward:	tttagttcctctctcagcagctgactcg	This study
CNAβ1_C493S quickchange Reverse:	cgagtcagctgctgagagaggaactaaa	This study
CNAα NIR>AAA quickchange Forward:	ctgcagtattgaagtatgagaacaatgttatggctgccgcgcaatctaactgttctcctcatccatactggc	Wigington et al., 2020 [1]
CNAα NIR>AAA quickchange Reverse:	gccagtatggatgaggagaacagttgaattgcgcggcagccataacattgttctcatacttcaatactgcag	Wigington et al., 2020 [1]

References

1 Wigington, C. P. *et al.* Systematic Discovery of Short Linear Motifs Decodes Calcineurin Phosphatase Signaling. *Mol Cell* **79**, 342-358 e312, doi:10.1016/j.molcel.2020.06.029 (2020).
