# Supplementary material for: Palmitoylation targets the calcineurin phosphatase to the phosphatidylinositol 4-kinase complex at the plasma membrane
Source: Nat Commun. 2021 Oct 18;12:6064. doi: 10.1038/s41467-021-26326-4 (PMC8523714; doi:10.1038/s41467-021-26326-4)
Supplement: Supplementary file 3 — Description of Additional Supplementary Files [file 41467_2021_26326_MOESM3_ESM.pdf]

Description of Additional Supplementary Files

File Name: Supplementary Data 1
Description: High-confidence interactome. SAINTexpress was run with default parameters as described in the Methods (SAINTexpress task 1858; P84, VS14). Prey Accession is the NCBI protein accession number; Prey Gene Symbol and Gene ID are as per NCBI Entrez Gene. Spectral counts for the prey (column E, separated by "I" delimiter; column F, summed, column G, averaged), number of replicates performed (column H), spectral counts for the prey across all negative controls (column I), Averaged probability across replicates (column J), maximal probability (column K), Fold Change (counts in the purification divided by counts in the controls plus small factor to prevent division by 0; column L) and Bayesian FDR (column M) are listed for each bait-prey relationship and are directly from the SAINTexpress output. Only high-confidence interactors (i.e. with SAINT BFDR ≤1% are listed. Data set is publicly available and was deposited to MassIVE database with accession code MSV000087664[https://massive.ucsd.edu/ProteoSAFe/dataset.jsp?task=d0f513c6ec37426e9a598ccd2a8137a9] In the MassIVE deposition, Table 3 contains complete SAINTexpress output, Table 2 contains complete peptide evidence and Table 1 contains the list of samples processed. Note that the controls samples were also used in StDenis et al., Mol Cell Proteomics, 2015 and St-Denis et al., Cell Reports, 2016). Manually removed cells:
CNAβ2_P00761;CNAβ1_P00761;CNAβTrunc_P00761;CNAβ2_56160531;CNAβ1_56160531;CNAβTrunc_56160
531;CNAβ2_ENSEMBL:ENSβTruncAP00000024146;CNAβ1_ENSEMBL:ENSβTruncAP00000024146;CNAβTrun c_ENSEMBL:ENSβTruncAP00000018229. Manually removed prey lines:
P00761;56160531;ENSEMBL:ENSβTruncAP00000024146;ENSEMBL:ENSβTruncAP00000018229
