# Supplementary material for: Palmitoylation targets the calcineurin phosphatase to the phosphatidylinositol 4-kinase complex at the plasma membrane
Source: Nat Commun. 2021 Oct 18;12:6064. doi: 10.1038/s41467-021-26326-4 (PMC8523714; doi:10.1038/s41467-021-26326-4)
Supplement: Supplementary file 5 — Reporting Summary [file 41467_2021_26326_MOESM5_ESM.pdf]

nature portfolio

Corresponding author(s): Martha S Cyert

Last updated by author(s): Sep 27, 2021

Reporting Summary

Nature Portfolio wishes to improve the reproducibility of the work that we publish. This form provides structure for consistency and transparency in reporting. For further information on Nature Portfolio policies, see our Editorial Policies and the Editorial Policy Checklist.

Statistics

For all statistical analyses, confirm that the following items are present in the figure legend, table legend, main text, or Methods section.

n/a	Confirmed	
☐	☒	The exact sample size (*n*) for each experimental group/condition, given as a discrete number and unit of measurement
☐	☒	A statement on whether measurements were taken from distinct samples or whether the same sample was measured repeatedly
☐	☒	The statistical test(s) used AND whether they are one- or two-sided<br>*Only common tests should be described solely by name; describe more complex techniques in the Methods section.*
☒	☐	A description of all covariates tested
☐	☒	A description of any assumptions or corrections, such as tests of normality and adjustment for multiple comparisons
☐	☒	A full description of the statistical parameters including central tendency (e.g. means) or other basic estimates (e.g. regression coefficient) AND variation (e.g. standard deviation) or associated estimates of uncertainty (e.g. confidence intervals)
☐	☒	For null hypothesis testing, the test statistic (e.g. *F*, *t*, *r*) with confidence intervals, effect sizes, degrees of freedom and *P* value noted<br>*Give P values as exact values whenever suitable.*
☐	☒	For Bayesian analysis, information on the choice of priors and Markov chain Monte Carlo settings
☒	☐	For hierarchical and complex designs, identification of the appropriate level for tests and full reporting of outcomes
☐	☒	Estimates of effect sizes (e.g. Cohen's *d*, Pearson's *r*), indicating how they were calculated

Our web collection on statistics for biologists contains articles on many of the points above.

Software and code

Policy information about availability of computer code

Data collection	Immunostained cell images were taken using Lionheart-FX, blots scanned using Licor Odyssey CLx or Typhoon Trio Scanner, plate reader used for BRET analysis performed using Varioskan Flash Reader, Impact HD QTOF Mass spectrometer, LTQ-Orbitrap Velos instrument
Data analysis	Images analyzed with Image J/Fiji (2.0) using EZcolocalization Plugin, data statistics performed using GraphPad Prism (9.1.0) and Excel (16.53), blots were analyzed using ImageQuant TL7.0 and ImageStudioLite (5.2.5), ClustalW alignments were performed in Jalview (2.11.1.4), Mass Spectrometry data analyses performed using Xcalibur 2.0 , HD-Examiner, ProteoWizard (3.0.4468), SAINTexpress, Mascot (2.3.02), Comet (2012.01 rev3), ProHits, and PEAKS7, NetPhos 3.1 database for protein phosphorylation , BioRender.com was used for schematics.

For manuscripts utilizing custom algorithms or software that are central to the research but not yet described in published literature, software must be made available to editors and reviewers. We strongly encourage code deposition in a community repository (e.g. GitHub). See the Nature Portfolio guidelines for submitting code & software for further information.

Data

Policy information about availability of data

All manuscripts must include a data availability statement. This statement should provide the following information, where applicable:

- Accession codes, unique identifiers, or web links for publicly available datasets
- A description of any restrictions on data availability
- For clinical datasets or third party data, please ensure that the statement adheres to our policy

The AP-MS data generated in this study have been deposited to the ProteomeXchange database through partner MassIVE under accession codes PXD026809 [http://proteomecentral.proteomexchange.org/cgi/GetDataset?ID=PXD026809] and MSV000087664 [https://massive.ucsd.edu/ProteoSAFe/dataset.jsp?task=d0f513c6ec37426e9a598ccd2a8137a9], respectively. The HDX-MS data generated in this study have been deposited to the ProteomeXchange database

through partner PRIDE under accession code PXD025900 [http://proteomecentral.proteomexchange.org/cgi/GetDataset?ID=PXD025900]. Uncropped and unprocessed scans of blots and quantifications as well as HDX-MS source data are provided in the Source Data file. The imaging data that was used to generate Figure 1e, f and Figure 2f, g and Supplementary Figure 1 e, f are published in Mendeley Data with DOI: 10.17632/85v4dj4kgm.1 . Structures of the calcineurin heterodimer and the PI4KA trimer, used in figure 4, are derived from the Protein Data Bank with accession codes 6NUC [https://www.rcsb.org/structure/6NUC] and 6BQ1[https://www.rcsb.org/structure/6BQ1] respectively.

Field-specific reporting

Please select the one below that is the best fit for your research. If you are not sure, read the appropriate sections before making your selection.

☒ Life sciences ☐ Behavioural & social sciences ☐ Ecological, evolutionary & environmental sciences

For a reference copy of the document with all sections, see nature.com/documents/nr-reporting-summary-flat.pdf

Life sciences study design

All studies must disclose on these points even when the disclosure is negative.

Sample size	No statistical methods were used to determine sample size prior to experiments. Sample sizes are given in the manuscript. The majority of biochemical assays were repeated at least three times in order to derive statistical information such as error bars, p values and significance. The number of cells analyzed for quantifications were determined based on previous experience and/or on previously published, similar experiments.
Data exclusions	No data were excluded from analyses.
Replication	All findings were reproduced independently at least three times unless otherwise noted in Methods or Figure legends. All microscopy images were analyzed for at least 70 cells across three independent replicates. All attempts of replication were successful.
Randomization	No randomization was found necessary as all experiments were conducted with appropriate positive and negative controls.<br>For imaging experiments, cells were chosen at random within each condition.
Blinding	Blinding was not considered to be necessary due to clear effects observed and because all samples in biochemical, blotting and imaging experiments were analyzed in exactly the same manner.

Reporting for specific materials, systems and methods

We require information from authors about some types of materials, experimental systems and methods used in many studies. Here, indicate whether each material, system or method listed is relevant to your study. If you are not sure if a list item applies to your research, read the appropriate section before selecting a response.

Materials & experimental systems

n/a	Involved in the study	
☐	☒	Antibodies
☐	☒	Eukaryotic cell lines
☒	☐	Palaeontology and archaeology
☒	☐	Animals and other organisms
☒	☐	Human research participants
☒	☐	Clinical data
☒	☐	Dual use research of concern

Methods

n/a	Involved in the study	
☒	☐	ChIP-seq
☒	☐	Flow cytometry
☒	☐	MRI-based neuroimaging

Antibodies

Antibodies used

Primary Antibodies:
Anti-Penta His antibody (1:2,000) QIAGEN Catalog # 34660; RRID: AB_2619735
Anti-GST antibody (1:1,000) BioLegend Catalog # 640802; RRID: AB_2189867
Anti-MYC antibody, clone 7D10 (1:2,500) Cell Signaling Catalog # 2278T; RRID: AB_490778
Anti-Beta-actin antibody (1:3,000) LiCOR Bio Sciences Catalog # 926-42210; RRID: AB_1850027
Anti-HA antibody (1:2,000) Sigma-Aldrich Catalog # H3663, Clone HA-7; RRID: AB_262051
Anti-FLAG antibody,clone M2 (1:2,500 in western) Sigma-Aldrich Catalog # F3165; RRID: AB_259529
Anti-FLAG, clone M2 (1:500 in immunofluorescence) Sigma-Aldrich Catalog # F1804, RRID:AB_262044
Anti-GM130, clone D6B1 (1:4000) Cell Signaling Technologies Catalog # 12480, RRID:AB_2797933
Anti-calnexin (1:3,000) Enzo Life Science Catalog # ADI-SPA-865, RRID:AB_10618434
Anti-Gapdh (1:20,000, clone 1E6D9, Proteintech Catalog # 60004-1-Ig, RRID:AB_2107436
Anti-GFP (1:4,000) Takara Bio Clontech Catalog # 632380, RRID:AB_10013427
Anti-RFP (1:3,000) Rockland Inc. Catalog # 22904

	Anti- Phospho-p44/42 MAPK (ERK1/2) (Thr202/Tyr 204), (1:3,000), Cell Signaling Technology Catalog # 4376, RRID:AB_331772<br>Anti- phospho FAM126A S485 (1:1,000) 21st Century Biochemicals<br>Secondary Antibodies<br>Anti-rabbit Brilliant Violet 421 (1:100) BioLegend Cat# 406410, RRID:AB_10897810<br>Streptavidin (Licor IRDye 800CW Streptavidin) LI-COR Biosciences Cat# 926-32230<br>Anti-mouse IgG1 Alexa 647 Thermo Fisher Catalog # A-21240, RRID: AB_141658<br>Anti-mouse IgG2b Alexa 488 Thermo Fisher Catalog # A11029, RRID: AB_138404
Validation	All primary antibodies are commercial antibodies, analyzed by immunofluorescence or immunoblotting and validated by the expected size and/or intracellular localization as described in each manufacturers` webpage.<br>Penta-His antibody validated by the vendor using immunoblots and immunohistochemistry. (https://www.qiagen.com/us/products/discovery-and-translational-research/protein-purification/tagged-protein-expression-purification-detection/anti-his-antibodies-bsa-free/?cmpid=PC_DA_NON_BIOCOMPARE_ProductListing_0121_RD_MarketPlace_ProductD)<br>Anti-GST antibody is validated by the vendor using immunoblotting in cell lysates expressing fusion proteins in detecting purified recombinant proteins . https://www.biolegend.com/en-us/products/purified-anti-gst-antibody-5160<br>Anti-MYC antibody is validated by the vendor using immunoblotting, indirect immunofluorescence and in flow cytometry analysis on lysates expressing fusion proteins. https://www.cellsignal.com/products/primary-antibodies/myc-tag-71d10-rabbit-mab/2278<br>Anti-Beta Actin antibody is validated by the vendor via westen blot, immunohistochemistry, immunofluorescence and flow cytometry.https://www.licor.com/bio/reagents/beta-actin-rabbit-monoclonal-antibody-for-normalization<br>Anti-HA antibody is validated by the vendor using immunoblotting, immunoprecipitation and immunofluorescence to detect fusion proteins. https://www.sigmaaldrich.com/deepweb/assets/sigmaaldrich/product/documents/444/447/h3663dat.pdf<br>Anti-FLAG antibody (#F3165) is validated by varios techniques including immunoblotting, immunoprecipitation, immunocytochemistry and ELISA by multiple publications linked in vendors webpage :https://www.sigmaaldrich.com/US/en/product/sigma/f3165?context=product<br>Anti-FLAG antibody (#F1804) is validated by the vendor to detect FLAG fusion proteins in immunoblotting, immunoprecipitation, immunofluorescence and immunocytochemistry in mammalian, plant and bacterial expression systems. https://www.sigmaaldrich.com/US/en/product/sigma/f1804?context=product<br>Anti-GM130 antibody is validated by the vendor via correct size in western blots from cell extracts and proper intracellular localization in mammalian cells. https://www.cellsignal.com/products/primary-antibodies/gm130-d6b1-xp-rabbit-mab/12480<br>Anti-Calnexin antibody is validated by the vendor via detection of correct size in western blots and immunocytochemistry. https://www.enzolifesciences.com/ADI-SPA-865/calnexin-polyclonal-antibody/<br>Anti-Gapdh is validated by the vendor to show reactivity in human, mouse, rat, yeast, plant and zebrafish using various applications including western blot, immunoprecipitation and immunofluorescence.https://www.ptglab.com/products/GAPDH-Antibody-60004-1-Ig.htm#product-information<br>Anti-GFP antibody is validated by the vendor using western blot analysis in cell lysates expressing GFP fusion protein as described in the vendors webpage:https://www.takarabio.com/documents/Certificate%20of%20Analysis/632380/632380-632381-070313.pdf<br>Anti-RFP antibody is validated by the vendor where it was used multiple applications including western blotting, immunoprecipitation and ELISA. https://rockland-inc.com/store/Antibodies-to-GFP-and-Antibodies-to-RFP-600-401-379-O4L_24299.aspx<br>Anti- Phospho-p44/42 MAPK (Erk1/2) is validated by the vendor using western blot and immunohistochemistry with approrpriate controls as described in the manufacturer`s page :https://www.cellsignal.com/products/primary-antibodies/phospho-p44-42-mapk-erk1-2-thr202-tyr204-20g11-rabbit-mab/4376<br>The custom developed Anti-phospho FAM126A S485 antibody is validated via immunoblotting and immunofluorescence in our own laboratory using recombinant, epitope-tagged human FAM126A (wildtype or with Serine 485 mutagenized) in human cell lines with appropriate controls and under different signaling conditions (see Figure 5).

Eukaryotic cell lines

Policy information about cell lines

Cell line source(s)	HEK293 Flp-In T-REx cells received from Prof. Anne-Claude Gingras, University of Toronto, Canada. Commerical source: Invitrogen<br>HeLa and HEK293T cells are gifts from Prof. Jan Skotheim, Stanford University, USA. Commercial source unknown.<br>Sf9 cells, Prof. John E. Burke, University of Victoria, Canada. Commercial source: Expression systems #94-001S<br>COS-7 cells, ATCC, Catalog # CRL-1651, RRID:CVCL_0224
Authentication	All cell lines are authenticated using PCR based STR profiling, results are available in Source Data file.
Mycoplasma contamination	Cell cultures are routinely tested for mycoplasma and tested negative for contamination.
Commonly misidentified lines<br>(See ICLAC register)	No commonly misidentified cell lines were used.
